# Genome-wide non-CpG methylation of the host genome during *M. tuberculosis* infection

**DOI:** 10.1038/srep25006

**Published:** 2016-04-26

**Authors:** Garima Sharma, Divya Tej Sowpati, Prakruti Singh, Mehak Zahoor Khan, Rakesh Ganji, Sandeep Upadhyay, Sharmistha Banerjee, Vinay Kumar Nandicoori, Sanjeev Khosla

**Affiliations:** 1 Centre for DNA Fingerprinting and Diagnostics (CDFD), Hyderabad 500001, India; 2 Graduate Studies, Manipal University, Manipal 576104, India; 3 Centre for Cellular and Molecular Biology (CCMB), Council of Scientific and Industrial Research (CSIR), Hyderabad, India; 4 Department of Biochemistry, School of Life Sciences, University of Hyderabad, Telangana State, India; 5 National Institute of Immunology, Delhi 110067, India

## Abstract

A mammalian cell utilizes DNA methylation to modulate gene expression in response to environmental changes during development and differentiation. Aberrant DNA methylation changes as a correlate to diseased states like cancer, neurodegenerative conditions and cardiovascular diseases have been documented. Here we show genome-wide DNA methylation changes in macrophages infected with the pathogen *M. tuberculosis*. Majority of the affected genomic loci were hypermethylated in *M. tuberculosis* infected THP1 macrophages. Hotspots of differential DNA methylation were enriched in genes involved in immune response and chromatin reorganization. Importantly, DNA methylation changes were observed predominantly for cytosines present in non-CpG dinucleotide context. This observation was consistent with our previous finding that the mycobacterial DNA methyltransferase, Rv2966c, targets non-CpG dinucleotides in the host DNA during *M. tuberculosis* infection and reiterates the hypothesis that pathogenic bacteria use non-canonical epigenetic strategies during infection.

Changes in the transcriptome of a cell closely parallel changes in its epigenome highlighting the pliability of the epigenome towards signals emanating from the environment. Amongst the various epigenetic modifications, DNA methylation is an important component of a mammalian cell’s epigenome^1^ and several studies have exemplified the dynamic nature of DNA methylation and its contribution in translating an environmental cue to a cellular response^2^. Interaction with an infectious agent also invokes a response from the infected mammalian cell that manifests as molecular changes including modulation of the epigenome. The susceptibility of a cell’s methylome to manipulation by an infectious agent has been reported. For example, *Human papillomavirus* protein E7 has been shown to directly associate with DNMT1 leading to aberrant methylation of host DNA^3^. DNA methylation changes have also been reported in response to the protozoan *Leishmania donovani* infection of human macrophages^4^. These epigenetic changes were found to suppress the host immune response, aiding the intracellular survival of the protozoan^4^.

*Mycobacterium tuberculosis*, an intracellular pathogen, that infects human macrophages is able to subvert the host defense mechanisms and can lay dormant for years^5^. Very little is known about the epigenetic changes that accompany infection of macrophages by *M. tuberculosis*. The mycobacterial protein Rv1988 has been shown to methylate histone H3 at R42 and repress gene expression^6^ whereas Rv3763 is involved in suppression of IFN-γ induced genes via TLR2, leading to histone hypoacetylation at CIITA promoter^7^. Previous work from our laboratory has shown direct interaction of Rv2966c with the macrophage epigenome and its ability to effect non-CpG methylation at specific genetic loci^8^. These studies indicated that *M. tuberculosis* might be interfering with host epigenetic response to aid in its survival. In the present study, we report global DNA methylation changes acquired upon infection of THP1 macrophages with the virulent *Mycobacterium tuberculosis* H37Rv. Using MBD (Methyl Binding Protein) protein based methylation-Seq assay, we identified around 23000 differentially methylated regions (DMRs) in infected macrophages. Methylation analysis of randomly selected genetic loci using MeDIP (Methylated DNA Immunoprecipitation) and bisulfite sequencing revealed these changes to be at cytosines present in non-CpG rather than the CpG dinucleotide context. These findings mirror our previous finding where the mycobacterial protein Rv2966c was found to be targeting non-CpG dinucleotides^8^ and reiterates the hypothesis that bacteria use non-canonical epigenetic strategies during infection^9^,^10^,^11^,^12^,^13^.

## Results

### *M. tuberculosis* infection causes genome-wide DNA methylation changes in THP1 macrophages

**Figure 1:**
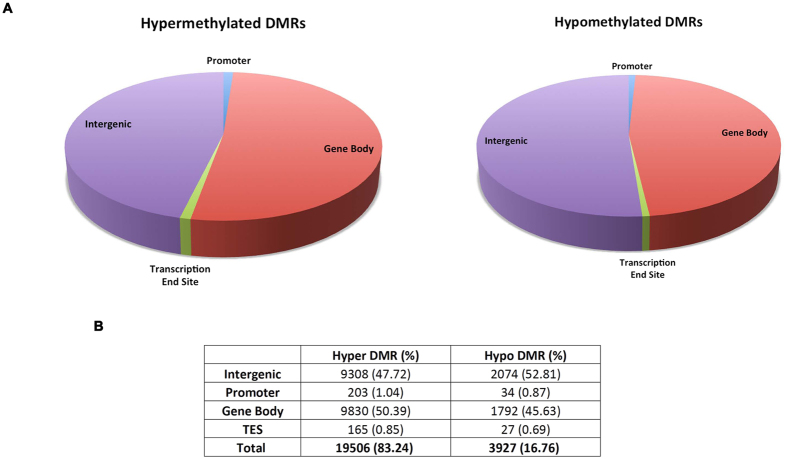
Genomic localization of the differentially methylated regions (DMRs) in *M. tuberculosis* infected THP1 macrophages. Pie charts (**A**) showing the distribution of DMRs (**B**) with respect to genes within the human genome. Hyper (left panel) and Hypo (right panel) DMRs were categorised as intergenic, promoter-specific and gene body specific transcription end site specific based on their location in the human genome. Hypermethylated DMRs represent genomic regions with gain of methylation while hypomethylated DMRs represent loss of methylation upon infection.

In order to study the DNA methylation dynamics during host-pathogen interaction, genomic DNA was isolated from *M. tuberculosis* H37Rv infected (0 and 48 hrs) and corresponding uninfected PMA treated THP1 cells (THP1 macrophages). DNA for each time point (in duplicates) was pooled and enriched for methylated regions by MBD protein based affinity pull down. The enriched DNA fragments were sequenced on the Illumina Hi-seq NGS platform (Materials and Methods). THP1 cell line, a monocytic cell line, differentiates into macrophages upon treatment with the mitogen Phorbol myristate acetate ester (PMA)^14^. In order to eliminate the DNA methylation changes that would have resulted due to differentiation, peaks that were different between uninfected 0 and 48 hrs samples were first removed. The remaining peaks were compared with peaks from infected 48 hrs samples (see flow charts in Supplementary Fig. 1). A total of 23,433 differentially methylated regions (DMRs) were identified, of which 19,506 (~83%) DMRs were hypermethylated and 3,927 (~17%) were hypomethylated in the *M. tuberculosis* H37Rv infected THP1 macrophages (Fig. 1). This indicated that regions of hypermethylation were five-fold more enriched than hypomethylation in the genome of infected cells.

### Genomic localization of the identified Differentially Methylated Regions

In order to assess the functional significance of the methylation differences arising due to *M. tuberculosis* infection, the distribution of these DMRs with reference to genic regions in the genome was examined. Approximately 50% of the hypermethylated DMRs (9830) were found to be associated with gene body (exons and introns), 48% (9308) were intergenic and 1% mapped to the promoter (203) and 1% to the transcription end site (TES, 165) (Fig. 1). In case of hypomethylated DMRs, ~46% (1792) and ~53% (2074) DMRs mapped to gene body and intergenic regions respectively while approximately 1% of DMRs were associated with promoter (34) and TES (27) (Fig. 1).

As 12946 intragenic DMRs (gene body, promoter and TES) were found to be associated with 7573 genes it indicated that multiple DMRs were associated with a single gene. Out of these 7573 genes, 4996 (3684 with hyper and 1312 with hypo-DMRs) were associated with a single DMR, 2555 (2278 with hyper and 277 with hypo-DMRs) with 2–10 DMRs and 22 (only hyper DMRs) with more than 10 DMRs (Table 1). Furthermore, out of the 7573 genes that were associated with a DMR, 2208 genes (listed in Supplementary Table 1) were found to be associated with both hypermethylated and hypomethylated DMRs. The epigenetic circuitry utilises DNA methylation to organize specific genetic loci into specific chromatin conformations^15^. The presence of both hypermethylated and hypomethylated DMRs within the same genetic loci could suggest multiple chromatin conformations within the same gene.

Analysis of DMRs mapping to the gene body revealed that DMRs were predominantly associated with introns as 93% of these DMRs mapped to the introns while only 4–5% mapped to the exons. The remaining DMRs were associated with 5′ or 3′ UTRs (Table 2). Amongst the DMRs mapping to the introns, 40% of DMRs mapped to the first two introns while ~12% mapped to the last two intron of a gene, irrespective of the gene size (Table 2). This observation was similar to what has been observed for B-cell methylome where MRIs (Methylated Region of Interest) were found frequently at 5′ and 3′ ends of a gene^16^.

**Figure 2:**
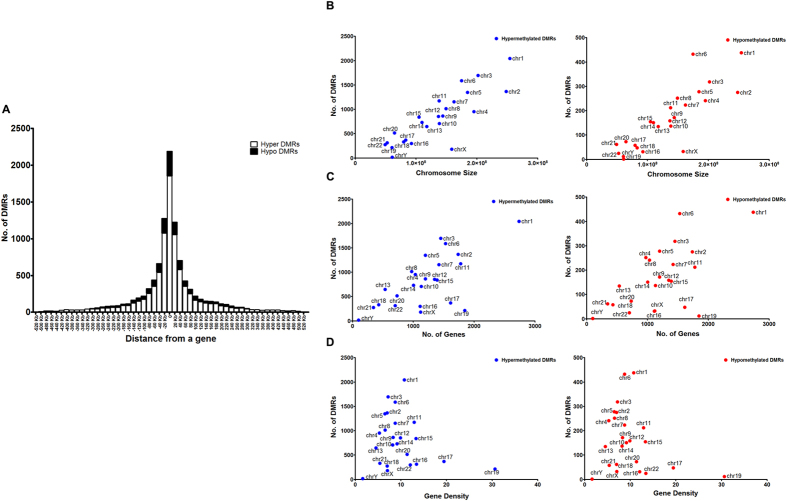
Characterization of DMRs. (**A**) Histogram showing distance of hyper (white bars) and hypo (black bars) DMRs from the transcription start site (TSS) of the nearest gene. Correlation of hyper (left panel) and hypo (right panel) DMRs with size (**B**), number of genes (**C**) and gene density (**D**) for each chromosome was plotted.

Examination of the intergenic DMRs showed that approximately 60% of the intergenic DMRs were present within 70 Kb of a TSS (52% for hypo and 62% for hyper-DMRs, Fig. 2A). Apart from regulatory elements, non-genic regions are generally associated with repetitive DNA sequences and ncRNA genes^17^. To examine if some of the DMRs were associated with any specific repetitive element, the percentage of a specific repetitive DNA element associated with the DMRs was compared with the percentage of this repetitive element within the whole genome (Table 3, see Materials and Methods). While SINE elements constitute only around 13% of the total length of the repetitive DNA sequence in the human genome, this percentage was significantly higher at approximately 33% in relation to the DMRs (Table 3). No major difference was observed for any other repetitive DNA elements.

ncRNAs including lncRNA, miRNA, piRNA and snoRNA are known to be associated with gene regulation^18^. Recent reports have also shown differential expression of host miRNA in response to *M. tuberculosis* infection^19^,^20^,^21^.In our study, approximately 8% (1850) of the DMRs were associated with non-coding RNA genes. Majority (~90%) of these DMRs were found to be intergenic. As many of the ncRNAs are present within or in the vicinity of protein coding genes^22^, it was no surprise that more than half of these DMRs (954) mapped within other genes. 61% of the ncRNA DMRs were associated with miRNA genes (1124), ~24% with lncRNA (452), ~8% with piRNA (156) and 6.4% with snoRNA (118) genes (Table 4).

### Chromosomal distribution of DMRs

In the human genome, distribution of genes on chromosomes has been found to be non-random and often in clusters. Studies have identified clusters of tissue-specific genes across different chromosomes^23^,^24^,^25^ that are co-expressed or co-regulated. To assess if these DMRs were preferentially associated with certain chromosomes and mirrored non-random chromosomal distribution of genes, distribution of the various DMRs on different chromosomes was examined (Supplementary Table 2).

**Figure 3:**
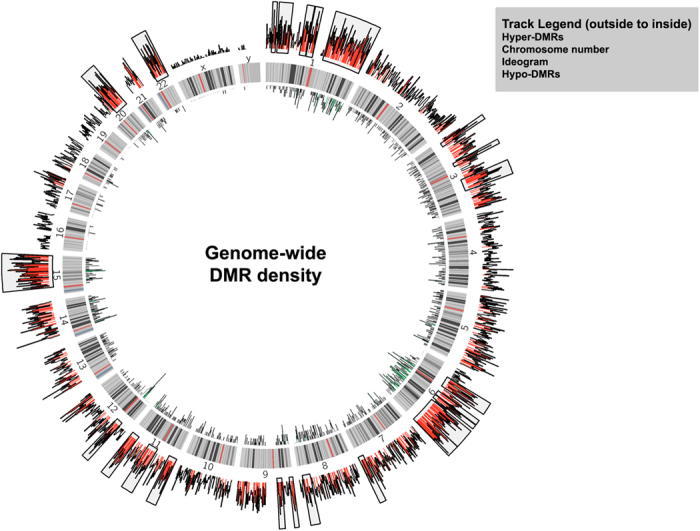
Identification of DMR hotspots. DMR density per Mb for each chromosome was calculated and plotted as histograms using Circos. The innermost circle displays the hypomethylated DMRs and the outermost circle displays the hypermethylated DMRs. Windows with more than 10 hypermethylated DMRs are shaded red and windows with more than 5 hypomethylated DMR are shaded green. DMR hotpsots within a chromosome are bound by a rectangle. The middle circle represents the ideogram. Bands drawn within the ideogram represent cytogenetic bands as are observed by Giemsa staining (data taken from UCSC Genome Browser).

While a positive correlation for both hypermethylated and hypomethylated DMRs was observed when compared to chromosome size as well as the number of genes per chromosome (Supplementary Table 2, Fig. 2B,C), no correlation was observed when gene density per megabase of chromosome was plotted against the number of DMRs, (Supplementary Table 2, Fig. 2D). The density of DMRs across the various chromosomes varied with chromosomes 17, 19 and 22 having high gene densities were found to have least number of DMRs. On the other hand, chromosome 1, 3 and 6 having low gene density had the highest number of DMRs (Fig. 3).

To further investigate the observed enrichment (within chromosomes 1, 3 and 6) and depletion (within chromosomes 17, 19 and 22) of DMRs, we calculated DMR density using a 5 Mb sliding window with a 500 kb slide. A hypermethylation hotspot was defined as any 5 Mb window with average DMR density higher than 38.47 (average DMR density for chromosome 6), the highest average DMR density observed amongst the 24 chromosomes (supplementary Table 3). Based on these criterion, 23 hotspots present on 11 different chromosome were identified (Fig. 3, Table 5). Chromosome 1 had the largest number of hotspots (5 nos.). A region on the long arm of chromosome 6 (6q21-6q27) containing several immunologically important genes (including *CCR6, TIAM2, ULBP2, LRP11, GPR126, FYN, NOX3*) was found to have the highest DMR density (Table 5, Supplementary Table 4).

### Functional classification of genes associated with DMRs

**Figure 4:**
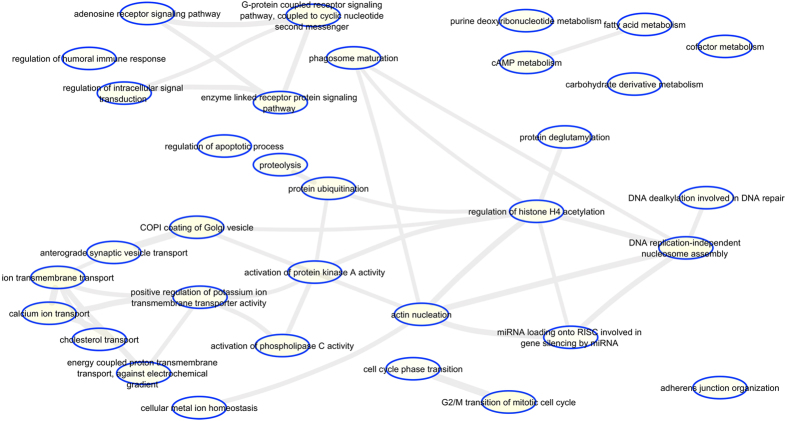
Gene Ontology (GO) enrichment analysis of genes. GO analysis of genes associated with gene body DMRs present within promoter, gene body and TES visualized as a REViGO Interactive Graph.

To examine if *M. tuberculosis* infection induced DNA methylation changes were associated with any specific genes or gene families, the genes associated with DMRs were classified using Gorilla Gene Ontology tool^26^ and the output was visualized using Revigo^27^. The gene families that showed significant enrichment (p < 0.05) included genes involved in signaling, cell communication, metabolism, transport, cell cycle, cytoskeleton reorganization, transcriptional regulation and chromatin modification (Fig. 4, Supplementary Table 5). DMR-associated immune response genes included genes corresponding to the HLA complex, cytokines, complement system.

**Figure 5:**
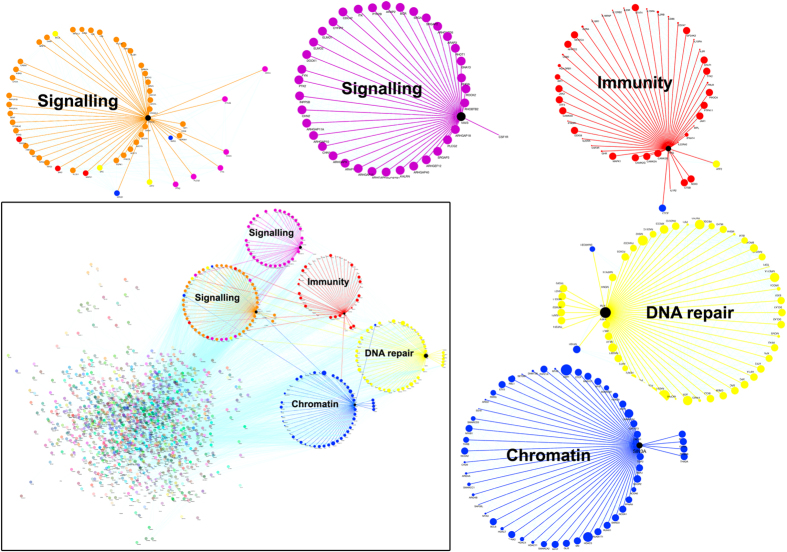
Protein network analysis. Network analysis was performed on the set of genes found to be in the vicinity of DMRs identified in the MBD-seq. The interaction between proteins was generated using STRING (high confidence) and output was visualized using Cytoscape (Bottom left panel). Prominent nodes with their first neighbors are highlighted as a circular layout. The prominent node identified were SIN3A, ATM, PRKCA, IFNG, VAV3. Zoomed images represent circular layouts depicting genes involved in signaling (orange and purple), immunity (red), DNA repair (yellow) and chromatin organization (blue).

The functional significance of genes found to be associated with regions of differential methylation in *M. tuberculosis* infected cells was also assessed by examining the interaction networks of the identified proteins. Using the web based PANTHER Gene Ontology tool^28^, we extracted the molecular function and biological process associated with each gene, followed by manual curation of the genes involved in immune response, chromatin modification, DNA replication and repair. The interaction network for the corresponding proteins was determined by the STRING search web tool^29^ and the interaction network was generated using clustering coefficient (Fig. 5). Most of the players involved in epigenetic reprogramming, were clustered in one sub-network. Interestingly, except for *DNMT1* (a maintenance methyltransferase), all other DNA methyltransferase genes, *DNMT3A, DNMT3B, DNMT3L,* were found to have *M. tuberculosis* infection related DMR(s). SIN3A, a protein known to be associated with HDAC mediated repression of MHC class II proteins and found to be associated with HLA-DRα promoter in *M. avium* infected THP1 cells^30^, formed an important node connecting immune response with the epigenetic machinery (Fig. 5). Other epigenetic modulators like DNA demethylases *TET2 and TET3,* histone variant genes, INO80 complex, PRC2 complex genes, *EED* and *SUZ12* were also associated with DNA methylation changes upon infection (Fig. 5).

**Figure 6:**
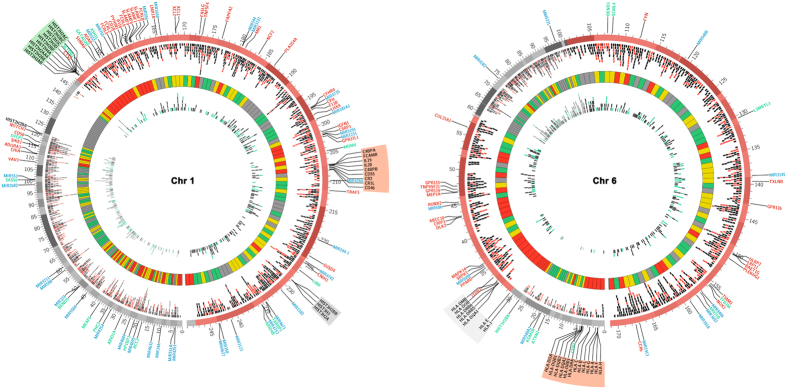
Circos plots for chromosome 1 and 6 highlighting DMR-associated features. Each DMR is plotted as a tile. The track immediately inside the ideogram represents hypermethylated DMRs, and the inner most track depicts hypomethylated DMRs. Hypermethylated and hypomethylated DMRs associated with the conserved sequence motif are colored red and green respectively. Regions within the chromosome identified as DMR hotspots are shaded red in the ideogram. Immune system related genes are labelled in red, ncRNA in blue and chromatin related genes in green. Hotspot cluster with histone genes is highlighted in green whereas the hotspots associated with HLA cluster on chromosome 6 and genes involved in immune response on chromosome 1 are highlighted in red. Histone and HLA genes that were not part of a hotspot are highlighted in grey. The heat map circle (second from inside) shows the gene density across the respective chromosome with regions of very high gene density as red, high as yellow, medium as green and low as grey.

We next examined the function of genes present within the top five hotspots to assess whether any particular gene(s) or gene family associated more prominently with the infection-induced differential DNA methylation (Table 5). It was interesting to note that several immunologically important genes were present within the 5 hotpots, (especially in hotspot 1, 3 & 4, Fig. 6, supplementary Table 4). Hotspot 1 present on chromosome 6 included immunologically important genes like *CCR6, TIAM2, ULBP2, LRP11, GPR126, FYN and NOX3* (Fig. 6). Hotspot 3 on chromosome 1 included *ADAR, FCRL2, IL20, CD46, CD55, TNFSF4, IGFN1, IL19, TRAF5*, etc, whereas hotspot 4 on chromosome 6 contained the HLA genes that are involved in antigen presentation apart from several other immunologically important genes (Fig. 6). The association of DMRs with histone genes (H2A gene cluster) was also noticeable in hotspot 3 on chromosome 1 (Fig. 6).

### DMRs are associated with a conserved motif

**Figure 7:**
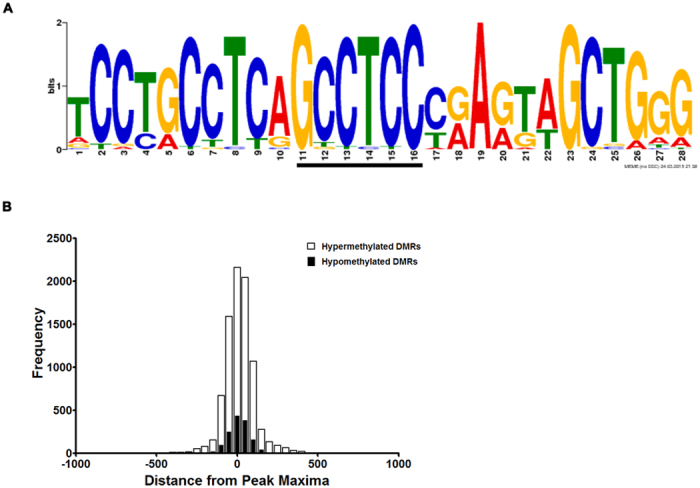
Identification of a common motif within DMRs. (**A**) The 28 bp motif identified in the DMRs and represented as a logo. The conserved ‘GCCTCC’ core has been underlined. The motif was generated using MEME tool. (**B**) Histogram showing the distance of the motifs, present within individual DMRs, from the peak maxima. Note that the maximum distance from the peak maxima for most of the DMRs was equivalent to the length of DNA wrapped around one nucleosome. Hyper DMRs are denoted by white bars and hypo DMRs with black bars.

To examine if a common motif was associated with the identified DMRs, the sequences corresponding to hypermethylated DMRs were tested for the presence of any conserved motif using MEME tool^31^. Since several immune response genes were present in the top 5 hotspots the analysis was initially performed on all the immune response genes associated DMRs. A 28 base pair motif with a conserved ‘GCCTCC’ core was identified in these DMRs using MEME (Fig. 7A). Based on this observation, the complete set of 23,433 DMRs were scanned for the presence of this motif. This motif was found to occur 8646 times in 6646 out of the 19,506 hypermethylated and 1455 times in 1178 out of the 3,927 hypomethylated DMRs (Supplementary Tables 6 and 7) with some DMRs showing the presence of this motif at multiple positions. In 515 genes this motif was present in both hypermethylated and hypomethylated DMRs (Supplementary Table 8). The enrichment of this motif in the human genome was also calculated. Taking the number of bases covered into consideration, this particular motif was more than 100 fold enriched in the DMR data set as compared to the whole genome. Scanning of individual chromosomes revealed that chromosome 19 had the maximum density of this motif. However, no significant enrichment of this motif was observed with any DMR across the different chromosomes (Supplementary Table 9). To further examine the correlation of this motif with DMRs, the distance of this motif from the peak maxima was calculated (Supplementary Table 10). In majority of the DMRs, the motif was found to occur within 150 bp from the peak maxima (Fig. 7B) and in 16% (1037) of hypermethylated and 20% of hypomethylated DMRs, the peak maxima and the motif overlapped (distance of 14 bp from the center of the 28 bp motif to peak maxima).

### *M. tuberculosis* infection induces methylation of non-CpG cytosines in host DNA

**Figure 8:**
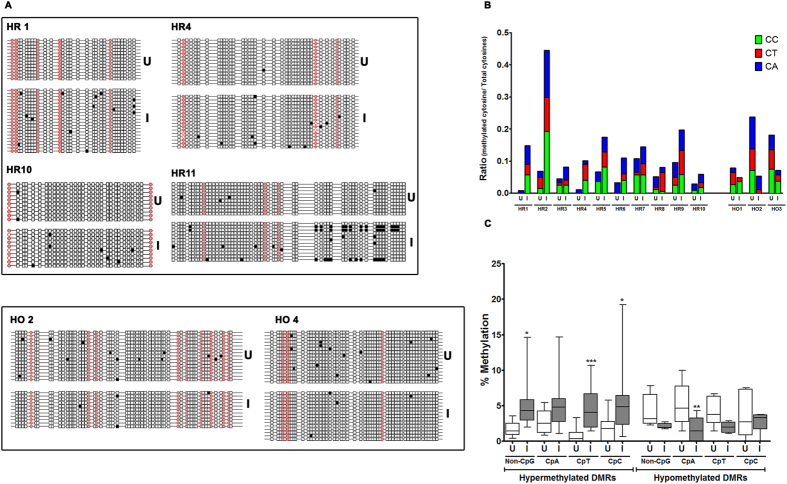
*M. tuberculosis* infection results in non-CpG methylation of the host genome. (**A**) DNA methylation analysis by bisulfite sequencing. Cytosine methylation profile for some of the hypermethylated (HR) and hypomethylated (HO) DMRs was examined by bisulfite sequencing of genomic DNA from uninfected (U) and *M. tuberculosis* infected (I) THP1 macrophages. Red circles represent CpG and squares represent non CpG dinucleotides. Filled symbols (grey or black) represent methylated cytosine. 10 or more clones per sample were analysed. (**B**) Statistical analysis of methylated non-CpG dinucleotides in hypermethylated (HR) and hypomethylated (HO) DMRs. Ratio of mC to total C per sample was plotted on Y-axis. HO2 - chr.3:28247576-28247639; HO4 - chr7:97993741-97993898; HR1 - chr6:167283490-167283654; HR4 - chr.3:162931192-162931429; HR10 - chr.5:39213638-39213737; HR11 - chr.1:164394535-164394664. (**C**) Percentage methylation of non-CpG dinucleotides. Proportion of methylated CpA, CpT and CpC dinucleotides was calculated with respect to total cytosines. The regions analysed are listed on the X-axis. Coordinates for each region are provided in Materials and Methods.

To validate the MBD-seq data and confirm DNA methylation changes observed in THP1 macrophages upon infection with *Mycobacterium tuberculosis,* we performed bisulfite sequencing on few of the DMRs. These DMRs were chosen either randomly or based on the role of the DMR associated gene in host defense mechanism against *M. tuberculosis* infection. Bisulfite analysis of these regions revealed negligible difference in methylation of cytosines present in CpG dinucleotide context. In fact, majority of the CpGs were found to be methylated in both uninfected and infected THP1 macrophages for the regions analysed (Fig. 8A, Supplementary Fig. 2). Surprisingly, a significant difference was obtained in the methylation of cytosines in non-CpG context for all the regions that we tested (Fig. 8A, B). This was true for both gain and loss of DNA methylation and the combined non-CpG methylation difference between uninfected and infected THP1 macrophages was also statistically significant (Fig. 8C). Examination of the bisulfite data also indicated that while change in the levels of cytosine methylation was observed in all the non-CpG dinucleotides, CpT methylation change was the most prominent (Fig. 8B,C).

**Figure 9:**
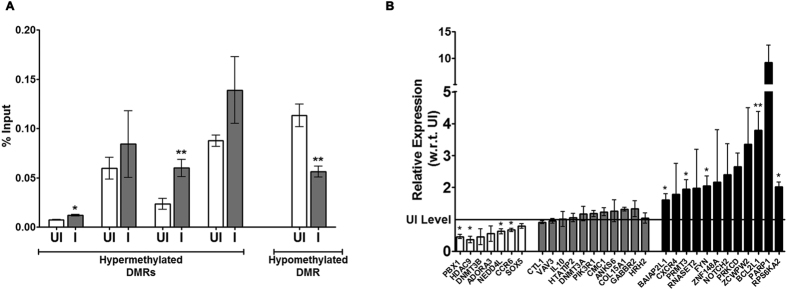
Infection associated DNA methylation changes are correlated with gene expression changes. (D) MeDIP validation of bisulfite sequencing based cytosine methylation enrichment of selected DMRs. The enrichment is represented as percentage input for each region. (E) Expression analysis by quantitative RT-PCR for the genes listed below the X-Axis. The level of expression in uninfected THP1 macrophages is shown by a horizontal line (UI level). The experiment was done at least thrice in duplicates. The error bars represent standard deviation (SD). * Indicate significant difference (Student’s t-test, *P < 0.05).

To confirm the genuineness of the observed non-CpG differential methylation by bisulfite analysis, methylation analysis was performed by MeDIP analysis on some of the DMRs examined by bisulfite sequencing. For this analysis, DNA was isolated from three different sets of THP1 macrophages infected with *M. tuberculosis* H37Rv. MeDIP results confirmed that the non-CpG methylation difference observed by bisulfite sequencing reflected the methylation difference that were observed initially by MBD-seq (Fig. 9A).

### Altered DNA methylation associated with gene expression changes

In order to study the effect of infection-induced DNA methylation changes on the expression levels of DMR-associated genes upon infection, expression of 28 genes associated with one or more DMRs was examined by Real Time RT-PCR. RNA for this analysis was isolated from three different sets of *M. tuberculosis* H37Rv infected or uninfected THP1 macrophages. 19 out of the 28 genes tested showed change in gene expression. 7 of them (*ADORA3, CCR6, DNMT3B, HDAC9, NEDD4L, PBX1, SOX5)* showed a decrease in expression, 12 genes showed increase in expression (*BAIAP2L1, BCL2L1, CXCR4, FYN, NOTCH2, PARP1, PRKCD, PRMT3, RNASET2, RPS6KA2, ZCWPW2, ZNF148*) while for 9 genes expression levels did not change (*ANKS6, CMC1, COL15A1, DNMT3A, FOXP2, GABBR2, HRH2, PIK3R1, VAV3*) 48 hours post-infection (Fig. 9B).

## Discussion

The dynamic nature of the epigenome allows a cell to translate environmental cues and mount appropriate response by modulating its transcriptional machinery. Here we show genome-wide DNA methylation changes upon infection of a macrophage by *Mycobacterium tuberculosis*. Importantly, the DNA methylation changes were non-canonical and observed only for cytosines present in non-CpG dinucleotide context.

Changes in DNA methylation upon *M. tuberculosis* infection was observed across the whole THP1 macrophage genome, on every chromosome and in proximity of 7573 genes. Although a few studies have shown correlation of gene body DNA methylation with gene regulation, most studies show a strong association of promoter DNA methylation with gene transcription^32^,^33^. It was, therefore, surprising to find that most of the DNA methylation changes upon *M. tuberculosis* infection were associated with non-promoter, non-exonic regions of the genome. Studies have shown that tissue-specific-methylated non-promoter regions are usually cis-regulatory elements like enhancers, transcriptional activators or repressors^34^. Therefore, the observation that DNA methylation predominantly at non-promoter regions was altered would indicate that regulatory regions were targeted by *M. tuberculosis* during infection of THP1 macrophages. This hypothesis is also supported by our finding that several ncRNA genes, which have been implicated in the regulation of gene expression^35^, were the target of infection-induced DNA methylation changes.

During different developmental stages or during cell differentiation, regulation of gene expression through DNA methylation is achieved both by gain and loss of DNA methylation at specific loci^36^,^37^. On a random basis, it is expected that the number of genetic loci gaining or losing DNA methylation should be equal. During *M. tuberculosis* infection of THP1 macrophages more than 90% of the differentially methylated regions showed gain of DNA methylation. The basis of DNA methylation gain could either be due to overexpression of host DNA methyltransferases like DNMT3A, DNMT3B or DNMT1 or down regulation of host DNA demethylases like TET proteins. Our preliminary data suggests that the expression levels of the known host DNA methyltransferases was not altered (Fig. 9B). Since changes in histone modifications are more dynamic than DNA methylation and the differentiated cells normally utilize the changes in histone modifications to initially respond to environmental cues^38^,^39^, it is possible that observed DNA methylation changes are secondary effect of changes in associated histone modifications. On the other hand, it is possible that the increase in DNA methylation was due to a mycobacterial protein that was secreted into the host by the pathogen. We have previously reported that the secretory mycobacterial protein Rv2966c can methylate the host genome^8^. This would indicate that the hypermethylation observed in macrophages upon mycobacterial infection could be due to the action of Rv2966c. Further work that dissects out the contribution of Rv2966c vis-a-vis a host DNA methyltransferase would help in understanding the reason for the bias towards hypermethylation during *M. tuberculosis* infection.

Mammalian DNA methyltransferases are known to methylate cytosines predominantly in CpG dinucleotides and the level of non-CpG methylation has been found to be very low or negligible in most differentiated cells^40^. Therefore, it was surprising to find that the cytosine methylation during *M. tuberculosis* infection of macrophages was predominantly at non-CpG dinucleotides. At some of the loci, while all CpG dinucleotides were fully methylated in both uninfected and *M. tuberculosis* infected macrophages, differential methylation between the two samples was solely due to methylation of cytosines in non-CpG dinucleotides. The mycobacterial protein Rv2966c has been shown to be a cytosine methyltransferase that predominantly targets non-CpG dinucleotides^8^. Therefore, our observation indicating non-CpG DNA methylation underscores the role of the mycobacterial protein Rv2966c in host DNA methylation upon *M. tuberculosis* infection. Non-CpG DNA methylation has been hypothesized to be removed only during replication^41^. Since macrophages, like other differentiated cells, do not undergo cell division, the acquired non-CpG DNA methylation and the associated chromatin changes at specific loci would be maintained in the *M. tuberculosis* infected macrophages. This would indicate that success of *M. tuberculosis* as a pathogen could partly be due to the non-canonical epigenetic mechanisms that it uses to modulate host gene expression.

While the differential DNA methylation was observed within or in the vicinity of genes belonging to diverse gene families we observed significant enrichment for a few gene families including immune response, chromatin modification, DNA replication and repair. Macrophages are antigen presenting cells and the prime target of infection by *M. tuberculosis*. Concordant with this fact, HLA genes, involved in antigen presentation and hence amplification of immune response, were found to be located within the DMR hotspot on chromosome six. Also targeted were the cytokine genes (*IL16, IL17*) and chemokine genes (*CCL17, CCL18*) that are secreted by activated macrophages and are essential for downstream regulation of T cell dependent immune response^42^,^43^,^44^,^45^. As indicated before, several micro RNAs, known to regulate gene expression, and lncRNA, known to be associated with chromatin modifying complexes like PRC2 and MLL, were found to be target of differential methylation. Our findings are in agreement with recent studies that have shown changes in the miRNA and lincRNA profile of TB patients and indicate that ncRNA along with important epigenetic effector molecules play an important role in the regulation of immunity-related genes during *M. tuberculosis* infection^20^,^21^,^46^.

In summary, our study shows that during *M. tuberculosis* infection, macrophages undergo genome-wide non-canonical DNA methylation changes that have the potential to modulate the host gene expression. However, further work is needed to understand the mechanism that underlie these DNA methylation changes and dissect out the role of specific mycobacteria proteins working alone or in concert with host epigenetic effectors in modulating host gene expression.

## Materials and Methods

### Infection of THP1 cells with *M. tuberculosis* H37Rv

PMA treated THP1 cells (ATCC) were infected with *M. tuberculosis* H37Rv as described elsewhere^8^ at an MOI of 1:1 or 1:10 (cells:bacteria) for 4 hrs followed by treatment with Gentamycin for 2 hrs. Cells were harvested after 0, 24 and 48 hours of infection and DNA & RNA were isolated using Qiagen All Prep Kit. For examining the efficiency of infection, dilution plating of mycobacteria harvested from infected THP1 cells, with and without gentamycin treatment, was done and colony forming units were counted at each time point (supplementary Fig. 3A). In order to assess the viability of infected THP1 macrophages, MTT assay was performed after 0, 24 and 48 hours of infection (Supplementary Fig. 3B). In addition, THP1 macrophages were also infected with PKH67 (Sigma) labeled *M. tuberculosis* H37Rv cells. Briefly 2 × 10^8^ mycobacterial cells were washed with PBS and resuspended in diluent C (Sigma). Cell suspension were then added to 2X dye and incubated at room temperature for 5 min. with periodic mixing. Reaction was quenched by addition of equal volume of serum, followed by three washes with PBS. Labeled cells were finally resuspended in 1 ml of RPMI and used for infection of THP1 macrophages. Infected cells were fixed after 0 and 24 hrs post-infection. The infected cells were visualized under confocal microscope (supplementary Fig. 3C).

MTT assay was performed as per manufacturer’s instructions. Briefly, cells were washed once with phenol free RPMI, followed by incubation with 1 mg/ml MTT for an hour at 37 °C. The cells were solubilized by addition of DMSO, formazan crystal was dissolved by titrating and absorbance measured at a wavelength of 540 nm. For CFU estimation of internalized bacteria, THP1 cells were lysed by addition of 0.05% SDS to release intracellular mycobacteria. 10 fold dilutions were plated on plain 7H11 plates and colonies were enumerated after 3–4 weeks of incubation.

The *M. tuberculosis* work was either performed in the P3 facility of National Institute of Immunology, Delhi, India or in the BSL-II type-II negative pressure facility within the laboratory of Dr. Sharmistha Banerjee, Department of Biochemistry, University of Hyderabad, Hyderabad, India as per approved IBSC guidelines.

### Methylated DNA pull-down using Methyl Miner kit

Genomic DNA isolated from uninfected and *M. tuberculosis* H37Rv infected THP1 macrophages was estimated using Qubit and DNA quality was checked by resolving on 0.8% agarose. Equal amount of DNA from two biological replicates was pooled and 3 μg of the pooled genomic DNA was fragmented using Covaris 2.0 in the range 150–180 bp. MBD-based methylated DNA pull down was performed on the fragmented DNA using Methyl Miner kit (Invitrogen) as per the manufacturer’s instructions. Enriched DNA was eluted twice using high salt buffer and precipitated. The efficiency of pull down was checked by end point PCR with *GAPDH* as negative control and a reported methylated region on Chr.22 as positive control (supplementary Fig. 4A,B). The eluted fractions were estimated by Qubit and fragment size checked by Bioanalyser (supplementary Fig. 4C).

### Library Preparation and Sequencing

Library Preparation was performed at Genotypic Technology’s Genomics facility (Bangalore, India) as follows. Libraries for multiplex ChIP Sequencing were constructed using NEXTflex™ ChIP-Seq Sample Preparation Kit protocol outlined in “Preparing Samples for ChIP Sequencing of DNA” (BIOO Scientific# IP-5143-01). Briefly, DNA was subjected to a series of enzymatic reactions that repair frayed ends and phosphorylated the fragments. The end-repaired fragments were adenylated with a single nucleotide ‘A’ overhang (BIOOScientific) followed by adapter ligation (NEXT Flex adapters). The fragments with ligated adapters were subjected to pre-size selection PCR for 5 cycles followed by size selection on 2% Low Melting Agarose. The size selected samples were enriched with 13 cycles of PCR. The prepared libraries were quantified using Nanodrop Spectrophotometer and Qubit followed by quality validation using High Sensitivity Bioanalyzer Chip (Agilent) (supplementary Table 11).

### Sequence Analysis and Peak calling

MBD libraries were sequenced on Illumina HiSeq platform at a depth of ~30 million paired end reads (100 t × 2). Only those reads which passed the Q30 filter were retained. We analyzed the quality of the sequences using FastQC (http://www.bioinformatics.babraham.ac.uk/projects/fastqc/), and mapped the short reads to the reference human genome (build hg19) using Bowtie2^47^, with a maximum mismatch of 2 bases. The alignment rate was >95% for all samples. We identified regions of significant enrichment using PeakSeq v1.1^48^ using Input as background control, tag extension of 200 bp, and a target false discovery rate (q value) of 0.01. The number of peaks obtained for each sample are outlined in Supplementary Table 1.

### Identification of Differentially Methylated Regions (DMRs)

To identify regions that showed a difference in their methylation levels upon *M. tuberculosis* H37Rv infection, a coverage based approach was followed. Firstly, for identification of regions hypermethylated upon infection, we took the peaks that were identified in I48 sample and calculated the coverage of these regions in all other samples using bedtools^49^. The coverage in each sample was normalized to its sequencing depth. Following this, all peaks that were smaller than 50 bp were discarded. To remove peaks that were significantly different between 0 and 48 hrs samples (probably due to cell culture) irrespective of infection, all regions that showed >25% difference in the coverage between U0 and U48 were removed. From the remaining regions, all peaks which had a coverage of less than 10 reads in I48 sample were removed to ensure that the coverage was significant to be called methylated. In addition, all regions that showed less than 50% increase in coverage in I48 with respect to U48 were also removed. To identify hypomethylated DMRs, the same approach was followed, except that we started with the peak-set obtained in U48, and the comparisons were done with respect to coverage of U48 instead of I48.

### Identification of hotspots

To identify potential hotspots of hypermethylated DMRs, the increase in the density (normalized for sequencing depth) for each DMR was calculated by subtracting the normalized density of a DMR in U48 from that of I48. The total increase for 500 kb sliding windows of 5 MB each was calculated for all chromosomes. A given 5 MB window was considered a hotspot if the DMR density in this window (per MB) was greater than 38.47, the average DMR density for chromosome 6, the highest average DMR density observed amongst the 24 chromosomes.

### Annotation of Peaks

For annotation, a non-redundant human gene database containing 28517 genes was created by retaining only the largest transcript of each official gene symbol. Each peak was annotated by mapping it to the nearest gene using bedtools, and further categorized the peaks into 4 groups – TSS (−1 kb to +0.5 kb of Transcription Start Site), TES (−0.5 kb to +0.5 kb of Transcription End Site), Gene Body (peaks present within the gene body but not TSS or TES, and intergenic peaks (all other peaks). Genic peaks were further annotated by mapping their peak maximas to 5′ UTR, CDS, Intron or 3′ UTRs.

### MeDIP

MeDIP was performed using Auto MeDIP Kit on automated platform SX-8G IP–Star Compact (Diagenode). Briefly, 1.5 microgram of DNA from uninfected and infected THP1 cells was sheared using Bioruptor to 200–500 bp. As per the manufacturer’s protocol, the sheared DNA was immunoprecipitated with 5-methylcytosine antibody, a portion of sheared DNA (10%) was kept as input and remaining immunoprecipitated DNA, bound to magnetic beads was isolated. qPCR for selected genomic locus using the primers listed below was performed and efficiency was calculated as %(me-DNA-IP/Total Input).


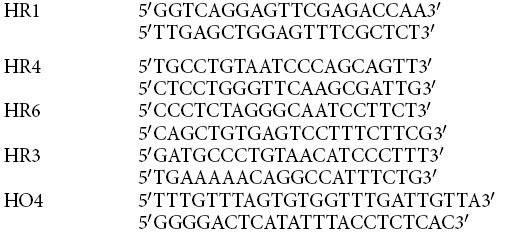


### Bisulfite Sequencing

Genomic DNA from uninfected and *M. tuberculosis* infected (48 hrs post-infection) THP1 macrophages was subjected to bisulfite conversion using Epitect Bisulfite Kit (Qiagen). PCR was performed on converted DNA using strand-specific modified primers. The PCR product was then cloned into XcmI digested pBSK-TA vector. At least 10 clones were analyzed for each sample.


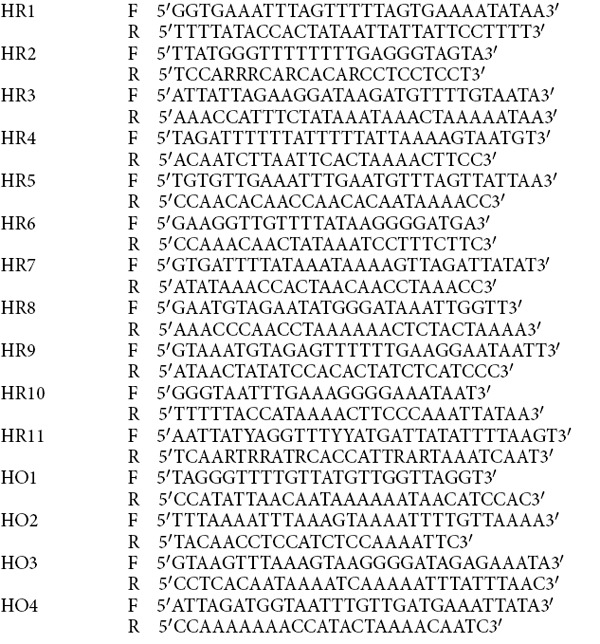


Hypomethylated


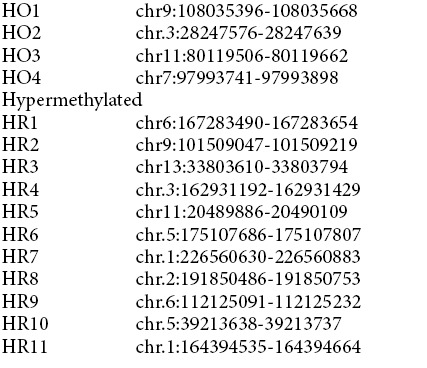


### Expression Analysis by Real Time PCR of *M. tuberculosis* infected THP1 macrophages

THP1 cells were infected with *M. tuberculosis* H37Rv as described above at an MOI of 10:1 for 4 hrs followed by treatment with gentamycin for 2 hrs. Cells were harvested at 0 hrs, 24 hrs and 48 hrs of infection and DNA-RNA were isolated using Qiagen All Prep Kit. 1 μg of RNA was converted to cDNA using SuperscriptIII (Invitogen). The change in expression upon infection of *M. tuberculosis* H37Rv in THP1 cells was evaluated by Real Time PCR using Mesa Green qPCR Mastermix Plus (Eurogentec) in ABI Prism SDS 7500 system. GAPDH was used as internal control. C_t_ values were normalized for GAPDH and fold change in infected sample with respect to uninfected samples was plotted.


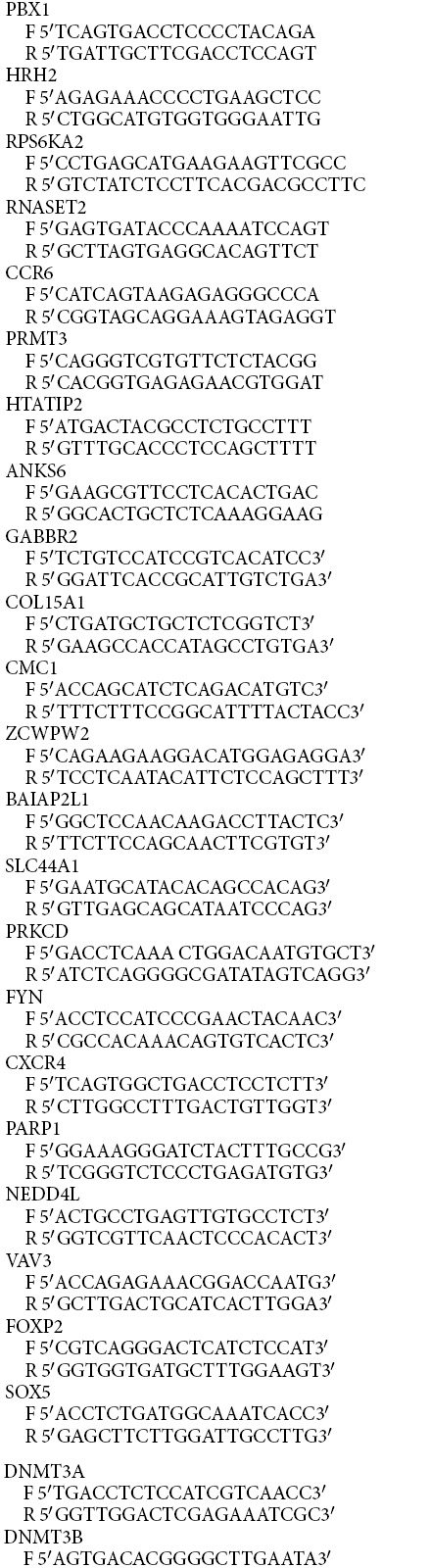


### Identification of motif using MEME tool

A text file containing chromosome number and the peak coordinates for DMRs associated with immune response genes was uploaded to Galaxy server to extract the sequences in fasta format. The same file was then uploaded to MEME suite to extract any conserved motif. The motif matrix obtained from the MEME output was then used to search for occurrence of that motif in the hypermethylated and hypomethylated DMRs. Occurrence of motif in DMRs was identified using FIMO.

### Identification and categorization of repeats in the DMRs

The association of DMRs with repeats was checked using a custom python script. Firstly, the repeat-masked sequence of all DMRs was extracted from the hg19 reference genome. For each DMR, the number of bases that were repeat-masked was calculated, and expressed as ratio to the total size of DMR. To further check if any given category of repeats was specifically associated with DMRs, the list of all repeat elements was downloaded using UCSC table browser^50^. Using this list, the number of bases of a DMR associated with a given repeat category was calculated. Specific enrichment of a repeat category was checked by comparing the fraction of DMRs associated with each category with the fraction of the entire genome associated with that category.

### Gene Ontology

The list of official gene symbol for genes associated with non-intergenic DMRs was submitted to GORILLA tools and functional overrepresentation test was performed to identify the enriched gene ontology (GO) categories^26^. The output was submitted to ReviGO^27^ and interactive graph was generated for selected GO terms. Using the web based PANTHER Gene Ontology tool (www.pantherdb.org), the molecular function associated with each gene was extracted and the genes were manually curated into the enriched GO categories.

The proteins involved in immune response, chromatin modification, DNA replication and repair were submitted to STRING (www.string-db.org). The interaction network so generated was extracted as a text file and submitted to Cytoscape (www.cytoscape.org) and clustering coefficient based circular layout algorithm was used to generate the network.

## Additional Information

**How to cite this article**: Sharma, G. *et al*. Genome-wide non-CpG methylation of the host genome during *M. tuberculosis* infection. *Sci. Rep.* **6**, 25006; doi: 10.1038/srep25006 (2016).

## Supplementary Material

Supplementary Information

Supplementary Table 1

Supplementary Table 2

Supplementary Table 3

Supplementary Table 4

Supplementary Table 5

Supplementary Table 6

Supplementary Table 7

Supplementary Table 8

Supplementary Table 9

Supplementary Table 10

Supplementary Table 11

## Figures and Tables

**Table 1:** Multiple DMRs associated with single genes.

No. of DMRs/ gene	Number of DMR-associated genes		
	Hyper DMR	Hypo DMR	Total
>10	22	0	22
2 to 10	2278	277	2555
1	3684	1312	4996
	**5984**	**1589**	**7573**

**Table 2:** Majority of the gene body DMRs are associated with introns.

Position of the DMR within a gene	Number of DMRs associated with Gene Body		
	Hyper DMR (%)	Hypo DMR (%)	Total (%)
Introns	9216 (92.5)*	1691 (93.1)*	10906 (92.6)*
First Intron	2211 (23.9)**	385 (22.8)**	2596 (23.8)**
Second Intron	1431 (15.5)**	294 (17.4)**	1725 (15.8)**
Penultimate	543 (5.9)**	104 (6.1)**	647 (5.9)**
Last Intron	595 (6.6)**	107 (6.3)**	702 (6.4)**
Other Introns	4436 (48.1)**	800 (47.4)**	5236 (48)**
Exons	462 (4.6)*	75 (4.1)*	537 (4.6)*
5′UTR	66 (0.7)*	8 (0.4)*	74 (0.6)*
3′UTR	221 (2.2)*	42 (2.3)*	263 (2.2)*

* Indicates w.r.t. gene body DMRs. **Indicates w.r.t. intronic DMRs.

**Table 3:** Repetitive DNA element associated DMRs.

Type of Repeat	Percentage of Repetitive DNA in the total Genome	Percentage of Repetitive DNA associated with DMRs	Hypo DMR
	hg19*	Hyper DMR	
LINE	21.57	19.84	20.78
SINE	**13.42**	**33.01**	**32.64**
LTR	9.05	7.27	7.09
Low_complexity	**0.58**	**0.14**	**1.81**
RC	0.02	0.02	0.04
Satellite	0.45	0.58	0.36
Simple_repeat	**0.89**	**1.58**	**3.10**
Other DNA repeats	3.49	3.03	4.19
RNA elements^1^	0.04	0.04	0.08
Unknown	0.04	0.04	0.06

Repeat elements showing preferential association with DMRs are in bold.

**Table 4:** ncRNA associated DMRs.

Type of ncRNA	No of ncRNA associated with DMR		
	Hyper	Hypo	TOTAL
miRNA	951	173	1124 (60.76%)
lncRNA	373	79	452 (24.43%)
piRNA	126	30	156 (8.43%)
snoRNA	93	25	118 (6.38%)
Total ncRNA	**1543**	**307**	**1850**

**Table 5:** Hypermethylation hotspots within the human genome linked to *M. tuberculosis* infection.

Chromosome No	Hotspot Start	Hotspot end	DMR density in the chosen window	DMR density in the Chromosome
chr6	101500000	171115067	54.4626	38.47
chr11	113500000	134000000	54.1656	33.03
chr1	149000000	248500000	52.0743	36.12
chr6	30500000	58000000	49.5417	38.47
chr1	105000000	121500000	48.2525	36.12
chr1	87500000	103500000	47.2857	36.12
chr3	7500000	18000000	47.2467	30.34
chr9	0	9500000	46.81	24.3
chr6	85500000	95500000	46.0091	38.47
chr1	29000000	58000000	44.8525	36.12
chr20	28000000	63025520	44.7732	31.38
chr1	15000000	25000000	44.4218	36.12
chr11	56000000	81500000	44.298	33.03
chr15	20500000	101500000	42.6521	31.11
chr3	115500000	146500000	42.4071	30.34
chr22	18500000	51304566	42.2277	25.37
chr7	145500000	159138663	42.0204	28.88
chr6	73500000	84500000	41.9733	38.47
chr12	20000000	36500000	41.7213	24.14
chr9	29000000	41500000	41.255	24.3
chr11	0	22000000	39.9337	33.03
chr3	68000000	77500000	39.134	30.34
chr8	117000000	137000000	38.8004	29.79
