# Supplementary material for: Genome-wide non-CpG methylation of the host genome during M. tuberculosis infection
Source: Sci Rep. 2016 Apr 26;6:25006. doi: 10.1038/srep25006 (PMC4845000; doi:10.1038/srep25006)
Supplement: Supplementary Information [file srep25006-s1.pdf]

Genome-wide non-CpG methylation of the host genome during *M. tuberculosis* infection

Garima Sharma[1,2#], Divya Tej Sowpati[3#], Prakruti Singh[1], Mehak Zahoor Khan[5], Rakesh Ganji[4], Sandeep Upadhyay[5], Sharmistha Banerjee[4], Vinay Kumar Nandicoori[5], Sanjeev Khosla[1*]

[1]Centre for DNA Fingerprinting and Diagnostics (CDFD), Hyderabad 500001, India [2]Graduate Studies, Manipal University, Manipal 576104, India.
[3]Centre for Cellular and Molecular Biology (CCMB), Council of Scientific and Industrial Research (CSIR), Hyderabad, India;
[4]Department of Biochemistry, School of Life Sciences, University of Hyderabad, Telangana State, India
[5]National Institute of Immunology, Delhi 110067, India.

Supplementary Figure 1

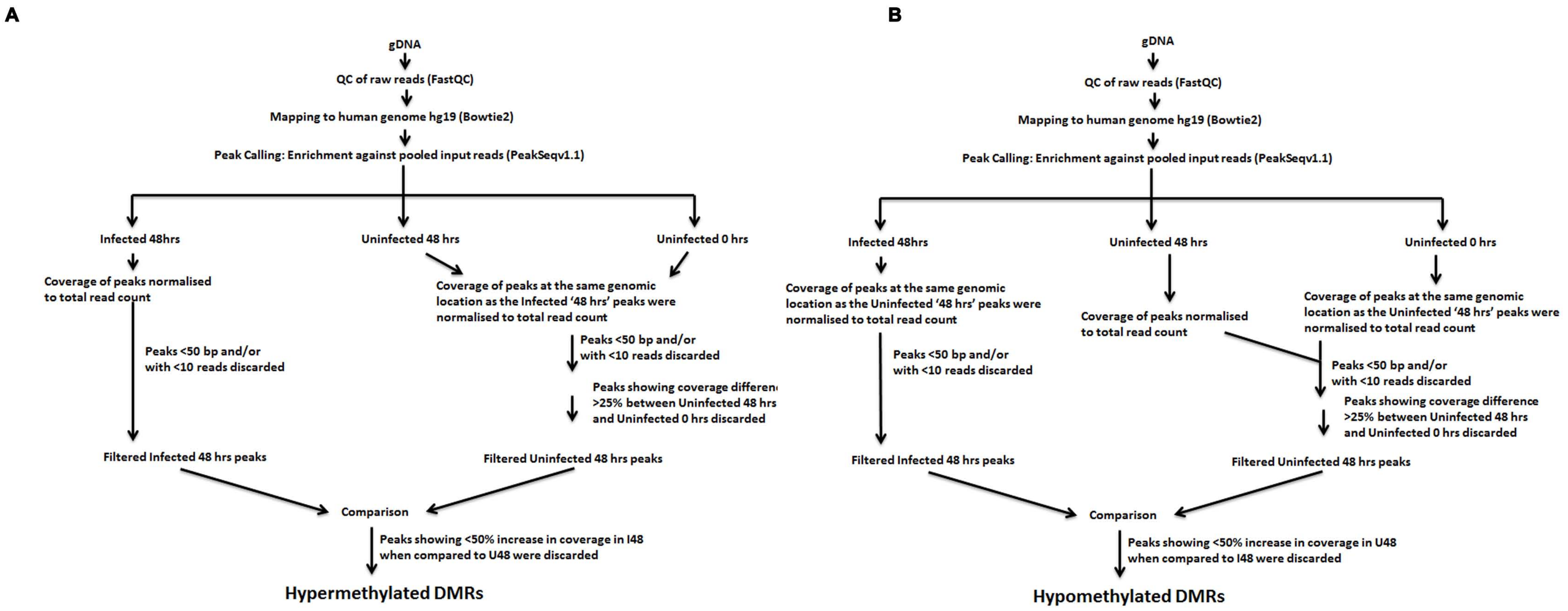

Supplementary figure 2

HR3

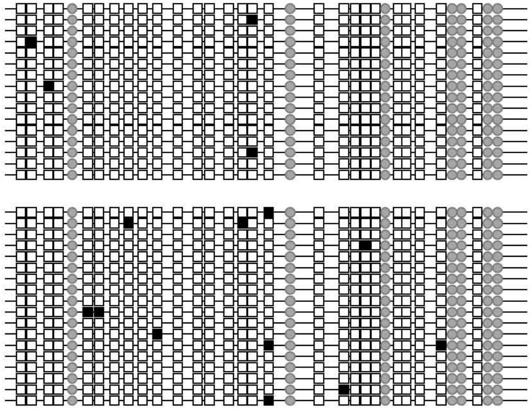

HR5

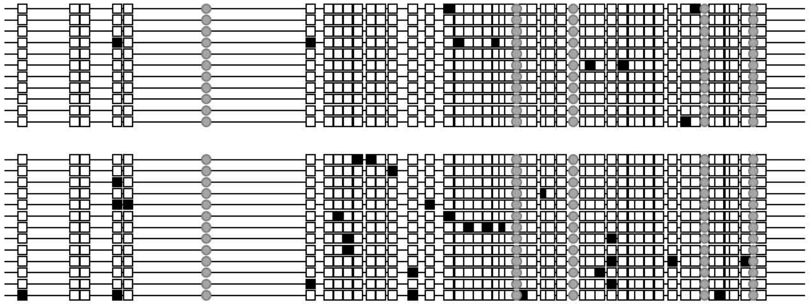

HR6

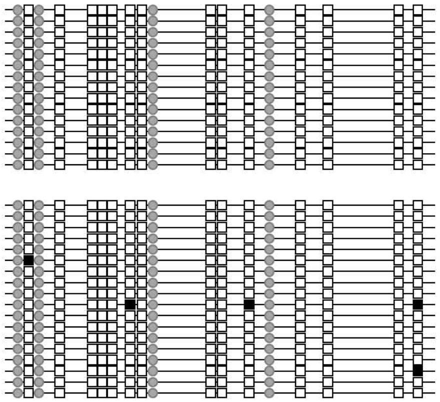

HR8

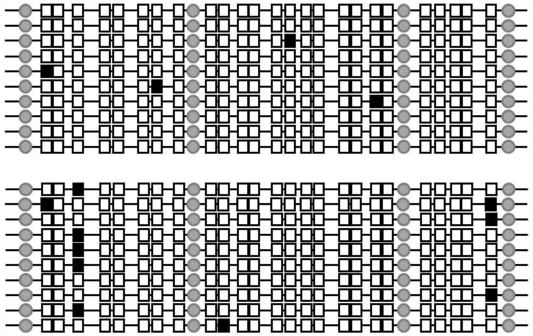

HR9

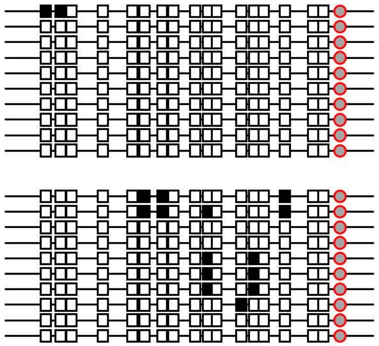

Supplementary Figure 3

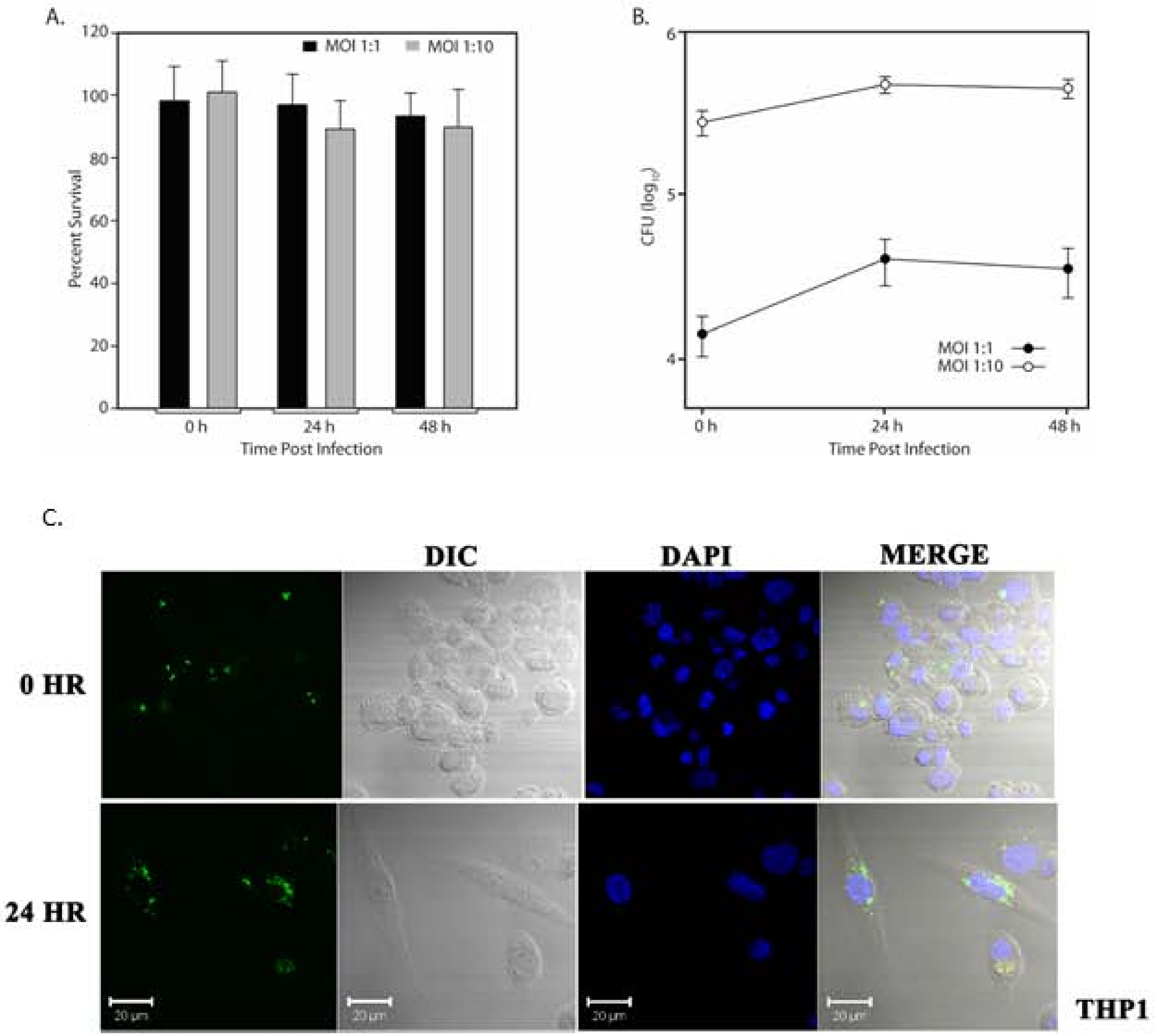

Supplementary Figure 4

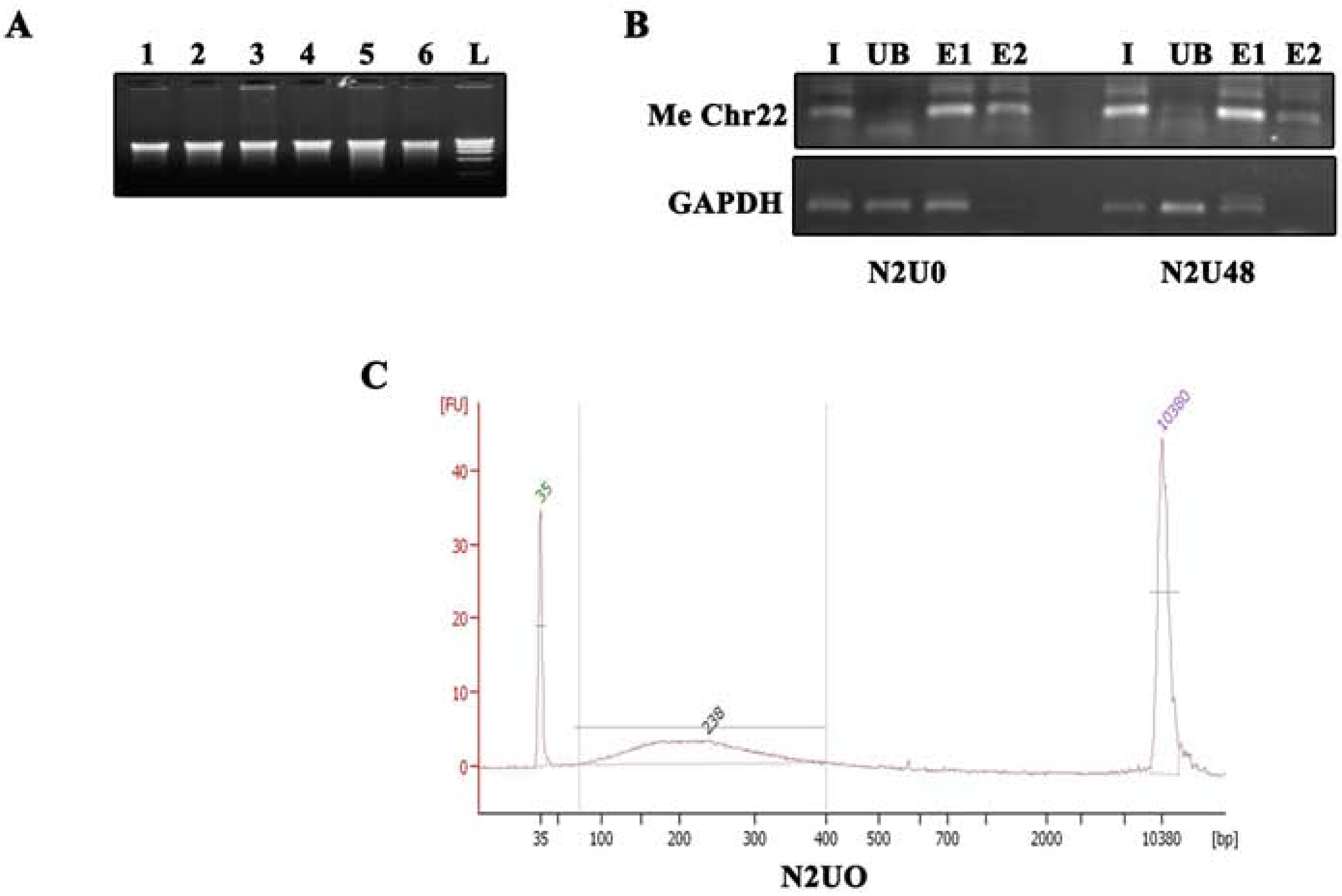

Supplementary information

Supplementary figure 1: Flow chart showing strategy for step-wise identification of the hypermethylated and hypomethylated DMRs.

Supplementary figure 2: Bisulfite sequencing analysis of the DMRs. Cytosine methylation profile of the hypermethylated (HR) and hypomethylated (HO) DMRs was analysed by bisulfite sequencing of genomic DNA from uninfected (U) and *M. tuberculosis* infected (I) THP1 macrophages. Red circles represent CpG and squares represent non-CpG dinucleotides. Filled symbols represent methylated cytosine. At least 10 clones per sample were analysed. HR3 - chr13:33803610-33803794; HR5 - chr11:20489886-20490109; HR6 - chr.5:175107686-175107807; HR8 - chr.2:191850486-191850753; HR9 - chr.6:112125091-112125232.

Supplementary figure 3: *M. tuberculosis* infection efficiency. A. MTT essay to demonstrate the viability of infected THP1 cells at M.O.I. of 1:1 and 1:10 (cells: bacteria) at different times points post infection (indicated below X-axis). B. CFU count for internalized *M. tuberculosis* bacilli at different time points post infection at the indicated M.O.I. C. THP1 macrophages infected with PKH67-labeled *M. tuberculosis* (green) at an MOI of 1:10 were visualised 0 24 hrs post-infection. The host nuclei are counter stained with DAPI (blue).

Supplementary figure 4: QC of gDNA and enriched me-DNA. A. Genomic DNA isolated from uninfected and infected THP1 macrophages was resolved on 0.8% agarose gel. Lane 1 to 3 - N2 sample; lane 4 -6 – N3 sample; Lane1-U0, 2-U48, 3-I48, 4-U0, 5-U48, 6-I48 respectively. L – λ/ Hind III DNA marker. B. Methylated DNA fragments enriched from THP1 macrophage gDNA using MBD were validated for pull-down efficiency by PCR using primers for a methylated region on chr22 (positive control) and GAPDH (negative control). Representative gel image for U0 and U48 is shown. I input, UB unbound, E elution. C: Bioanalyser output to assess the fragment size of N2U0 elution.
